# Hsa_circ_0008309 May Be a Potential Biomarker for Oral Squamous Cell Carcinoma

**DOI:** 10.1155/2018/7496890

**Published:** 2018-09-23

**Authors:** Bowen Li, Feng Wang, Xiang Li, Shuai Sun, Yuehong Shen, Hongyu Yang

**Affiliations:** ^1^Department of Oral and Maxillofacial Surgery, Peking University Shenzhen Hospital, Shenzhen, Guangdong 518036, China; ^2^Department of Oral and Maxillofacial Surgery, Sun Yat-sen Memorial Hospital, Sun Yat-sen University, Guangzhou 510120, China

## Abstract

**Objective:**

Oral squamous cell carcinoma (OSCC) is the most common cancer of the head and neck region. The circular RNA (circRNA) is known to serve an important role in the carcinogenesis of different types of cancer. However, the circRNA role of OSCC remains unclear.

**Methods:**

8 pairs of OSCC tissues and adjacent normal tissues were obtained to detect circRNAs expression by high-throughput sequencing, and 45 pairs of OSCC tissues were selected to verify the differentially significant circRNAs by reverse transcription-quantitative polymerase chain reaction (RT-qPCR). To further investigate the role of hsa_circ_0008309, the circRNA-microRNA (miR)-mRNA network was predicted using bioinformatics databases. The expression levels of hsa_circ_0008309, miR-1290, miR-136-5P, and miR-382-5P in SCC-15 and CAL27 cell lines were detected by RT-qPCR. Western blotting was performed to detect the protein level of Ataxin 1 (ATXN1).

**Results:**

The high-throughput sequencing results demonstrated that circRNAs were abundantly expressed in OSCC, and 16 circRNAs were significantly differentially expressed. Hsa_circ_0008309 was significantly downregulated in 45 pairs of OSCC tissue samples and was statistically correlated with pathological differentiation. The bioinformatics databases suggested that hsa_circ_0008309 could combine with miR-1290, miR-136-5P, and miR-382-5P, respectively, to regulate the expression of ATXN1. It was subsequently identified that hsa_circ_0008309 may inhibit miR-136-5P and miR-382-5P expression and increase ATXN1 expression in the OSCC cell lines.

**Conclusion:**

In summary, the results of the present study revealed that OSCC tissues have abundant circRNAs and, to the best of our knowledge, we firstly explore the regulatory role of the hsa_circ_0008309-miR-136-5P/hsa-miR-382-5P-ATXN1 network in OSCC. The results indicated that hsa_circ_0008309 may be a potential biomarker for OSCC.

## 1. Introduction

Oral squamous cell carcinoma (OSCC), the most frequently occurring oral malignancy, is the sixth most prevalent cancer worldwide and the third most common carcinoma in some developing countries [1, 2]. OSCC is one of the most frequently occurring head-neck tumors and accounts for ~80% of all cases [3]. In addition, ~600,000 new clinical cases are diagnosed annually and the age at the time of diagnosis is becoming lower each year [4]. Although the treatments modalities have improved, the mortality rate has not decreased significantly and the 5-year survival rate of OSCC is only ~63% [5]. Cancers including OSCC have been widely regarded as gene-related diseases; however, the precise molecular mechanisms and genetic basis of OSCC carcinogenesis remain largely unclear. Therefore, it is imperative to identify therapeutic targets to improve OSCC diagnosis and treatment.

Recently, circular RNAs (circRNAs) have been considered to be a special type of noncoding RNA, which are widespread and diverse in mammals [6]. Unlike linear RNA, circRNA contains covalently closed loop structures and has neither 5'caps nor 3'tails; therefore, the majority of circRNA cannot be degraded by RNA exonucleases or RNase R, thereby maintaining an improved stability compared with linear transcripts [6]. Emerging evidence has revealed various functions of circRNA such as competing endogenous RNAs, miRNA sponges, or transcriptional regulators [7]. CircRNA has also been considered to serve a crucial role in cancers, and due to its functions, circRNAs may serve as a potential target for tumor therapy or diagnostic biomarkers [8]. For example, CDR1as and circ-FOXO3 sponge microRNA (miR) regulate the development of breast carcinoma; additionally, circ-ITCH has an inhibitory effect on esophageal squamous cell carcinoma by suppressing the Wnt/*β*-catenin pathway [9–11]. However, the role of circRNA in the initiation and progression of OSCC has not been completely elucidated. In the present study, circRNA expression profiles were analyzed through high-throughput sequencing, followed by RT-qPCR to verify the significantly expressed circRNAs in the OSCC tissues. Hsa_circ_0008309 was identified to be downregulated in the cancer tissues. In order to explore the functions of hsa_circ_0008309, the hsa_circ_0008309 miR binding sites were predicted, and closely associated circRNA-miR-mRNA networks were constructed using bioinformatics approaches. These networks were further investigated in OSCC cell lines. In summary, the data provided a novel basis for circRNA functional research in OSCC and indicated that hsa_circ_0008309 may be a novel potential biomarker in OSCC research.

## 2. Materials and Methods

### 2.1. Patients and Samples

A total of 45 pairs of frozen OSCC tissues and adjacent normal tissues were acquired from patients with OSCC and these tissues were prepared for high-throughput sequencing and validation by RT-qPCR. These samples were acquired from the Department of Oral and Maxillofacial Surgery of Peking University Shenzhen Hospital (Shenzhen, China) from June 2015 to June 2016. Patients had not undergone additional treatments prior to surgery and all OSCC tissues were confirmed by strict pathological examination. The age of patients ranged from 29–78 years, and the median age at the time of diagnosis was 54 years. The male to female ratio was 34 : 11. Tissues were obtained from the tongue, gingiva, bucca, and floor of the mouth. Clinical and pathological characteristics of patients were based on the most recent World Health Organization (WHO) classification and UICC tumor-node-metastasis (TNM) classification [12]. Written informed consent from all patients was obtained, and the study was approved by the Medical Ethics Committee of Peking University Shenzhen Hospital. All methods were performed in accordance with the relevant guidelines and regulations.

### 2.2. RNA Extraction

Samples obtained from surgical specimens were immediately frozen using liquid nitrogen. Total RNA was extracted from frozen tissues using TRIzol® reagent (Thermo Fisher Scientific, Inc., Waltham, MA, USA) according to the manufacturer's protocol. The quality and quantity of the RNA were evaluated at a 260/280 ratio using a NanoDrop spectrophotometer (Thermo Fisher Scientific, Inc.).

### 2.3. Library Construction and High-Throughput Sequencing

Following extraction, total RNA was treated with RNase R to degrade the linear RNA and purified with RNeasy MinElute Cleanup Kit (Qiagen, Inc., Valencia, CA, USA). Next, a strand-specific library was constructed with VAHTS Total RNA-seq (H/M/R) Library Prep Kit for Illumina according to the manufacturer's protocol. In brief, ribosomal RNA was removed to retain the circRNAs. The enriched circRNAs were broken into short fragments using a fragmentation buffer and reverse transcribed into cDNA with random primers. Secondly, strand cDNA fragments synthesized by DNA polymerase I were purified with VAHTSTM DNA Clean Beads and liquated to Illumina sequencing adapters. Uracil-N-glycosylase was used to digest the second-strand cDNA. The digested products were purified with VAHTSTM DNA Clean Beads, amplified, and sequenced with Illumina HiSeq™ 2500 by Gene Denovo Biotechnology Co. (Guangzhou, China). The edgeR package (http://www.rproject.org/) was used to identify differentially expressed circRNAs. Some significant circRNAs were blasted against the circBase for annotation [13]. The circRNAs that could not be annotated were defined as novel circRNAs.

### 2.4. RT-qPCR

The reaction mixture (20 *μ*l) containing 1 *μ*g of total RNA was reverse transcribed into cDNA with the PrimeScript RT Master Mix (Takara Biotechnology Co., Ltd., Dalian, China). The mixture was incubated at 37°C for 15 min and 85°C for 5 sec to acquire cDNA. RT-qPCR was conducted with a Roche Applied Science LightCycler® 96 Real-Time PCR System (Roche Diagnostics, Indianapolis, Indiana, USA) in accordance with the manufacturer's protocol. The reaction mixture comprised of 2 *μ*l cDNA, 5 *μ*l SYBR® Premix Ex Taq™ II (Takara, China) and 1 *μ*l primers (reverse and forward) with RNase-Free water to a final volume of 10 *μ*l. The thermocycling conditions were as follows: 95°C for 3 min and amplified by 40 cycles of denaturing at 95°C for 10 sec and 60°C for 30 sec. *β*-actin was used as an internal standard. Melting curves were produced to check product purity and the expression levels of circRNAs were detected by using the 2^-*Δ*ΔCq^ method. The Cq value was the fractional cycle number at which the fluorescence exceeded the given threshold [14]. Primer sequences are listed as follows: hsa_circ_0008309 forward primer: 5′-ACAGCTATGGTGATGATTAGAGACA-3′; hsa_circ_0008309 reverse primer: 5′-TCAGAAGGTCCCAAATGCTGTT-3′; *β*-actin forward primer: 5′-AAACTGGAACGGTGAAGGTG-3′; *β*-actin reverse primer: 5′-AGTGGGGTGGCTTTTAGGAT-3′.

### 2.5. Prediction for the CircRNA-miRNA-mRNA Interaction

To investigate hsa_circ_0008309 function, the circRNA-miR-mRNA network was theoretically predicted through DIANA (http://diana.imis.athena-innovation.gr), MiRanda (http://www.microrna.org), and TargetScan (http://www.targetscan.org) databases. According to conserved seed-matching sequence principles, the predicted miRs and potential target genes were chosen by identifying the intersection of three databases [15]. The graph of the circRNA-miR-mRNA network was visualized by Cytoscape 3.01 (http://www.cytoscape.org).

### 2.6. Vector Construction

In order to produce the hsa_circ_0008309 transcript formation *in vitro* by nonlinear splicing, we constructed the hsa_circ_0008309 overexpression vector. The hsa_circ_0008309 linear sequence was synthesized and added to the pLCDH-ciR vector (Geneseed Biotechnology Co., Guangzhou, China) for the circularization of transcripts. The front circular frame contained the endogenous flBiotec genomic sequence with an *Eco*RI restriction enzyme site, and the back circular frame contained part of the inverted upstream sequence with a *Bam*HI site. The cDNA encoding hsa_circ_0008309 was amplified using primers 5′-CGGAATTCTGAAATATGCTATCTTACAGATGACCATGGATGAAAAATATGTA-3′ and 5′-CGGGATCCTCAAGAAAAAATATATTCACCATGTACATTAGTATGTCTCTA-3′ in the 293T cell line (Geneseed Biotechnology Co.). As a result, the amplified fragment was cloned into the vector between the two reading frames, and the mock vector was confirmed to contain a nonsense sequence between the two circular frames without the hsa_circ_0008309 encoding cDNA. The result of vector construction was verified by direct sequencing. The vectors were constructed with the help of Guangzhou Geenseed Biotech Co.

### 2.7. Cell Culture and Plasmid Transfection

The 293T cell line (Geneseed Biotechnology Co.) and SCC15 and CAL27 (purchased from the American Type Culture Collection, Manassas, VA, USA) cell lines were cultured in Dulbecco's modified Eagle's medium supplemented with 10% fetal bovine serum (both from Gibco; Thermo Fisher Scientific, Inc.), and culture plates were incubated at 37°C in a 5% CO_2_ humidified incubator. The empty vector without the hsa_circ_0008309 encoding cDNA was used as negative control. According to the manufacturer's protocol, hsa_circ_0008309 overexpression vector, miR-136-5P inhibitor plus hsa_circ_0008309 expression plasmid, miR-382-5P inhibitor plus hsa_circ_0008309 expression plasmid, and an empty control vector was, respectively, transfected into cell lines with 4000 ng plasmids using Lipofectamine 3000 (Invitrogen; Thermo Fisher Scientific, Inc.), and cell lines were harvested at 24 h following transfection.

### 2.8. Western Blotting

Cells were lysed with Blue Loading Buffer Pack (Cell Signaling Technology, Inc.) with a protease inhibitor cocktail and phenylmethanesulfonyl fluoride (Cell Signaling Technology, Inc.). The protein concentrations were quantified using a BCA Protein Assay Kit (Cell Signaling Technology, Inc.). Total cellular proteins (20 *μ*g) were separated via 10% SDS-PAGE and transferred to a polyvinylidene difluoride membrane (Millipore). Bovine serum albumin (BSA; 5%) was used as a blocking agent to reduce background and nonspecific binding. After blocking for 1 h, the membranes were incubated overnight at 4°C with rabbit monoclonal anti-human Ataxin 1 (ATXN1) and rabbit monoclonal anti-human GAPDH (both 1 : 1000; both from Cell Signaling Technology, Inc.) antibodies. GAPDH was used as a loading control for normalization. Following intensive washing, the membranes were incubated with anti-rabbit horseradish peroxidase-conjugated secondary antibodies (1 : 1000, Cell Signaling Technology, Inc.) for 1 h at room temperature. The protein bands were visualized using SignalFire™ ECL Reagent with the ImageQuant LAS4000 system (Fujifilm, Tokyo, Japan).

### 2.9. Statistical Analysis

All experiments were repeated three times, and data were presented as the means ± standard deviation. Differences and characterizations in circRNA expression profiles between OSCC tissues and adjacent noncarcinoma tissues were assessed by Pearson's correlation test. Hsa_circ_0008309 expression level between OSCC tissues and para-cancerous tissues was evaluated by two-tailed Student's *t*-test. The Student's *t*-test (two-tailed) was performed to analyze the association between the hsa_circ_0008309 expression level and the clinicopathological features of patients with OSCC. A one-way analysis of variance test was used to analyze hsa_circ_0008309 expression between different pathological groups, and the post hoc test was Tukey's multiple comparison test. Correlations between the circRNA expression level and miRNAs were evaluated by one-way ANOVA and Tukey's multiple comparison test. The clinical diagnostic value of hsa_circ_0008309 was verified by receiver operating characteristic (ROC) curve analysis in which an area under the curve (AUC) = 0.5 indicated no diagnostic value. *P* < 0.05 was considered to indicate a statistically significant difference. All statistical analyses were performed by GraphPad Prism 5.0 (GraphPad Software, La Jolla, CA, USA).

## 3. Results

### 3.1. Profile of Differentially Expressed CircRNAs in Patients with OSCC

**Figure 1:**
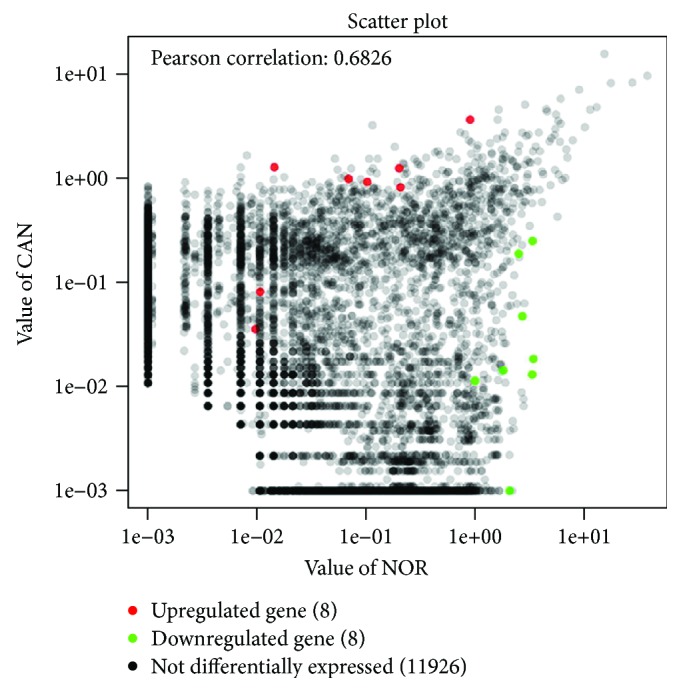
Scatter plot illustrating the expression profile of 11,942 circRNAs between the OSCC tissue and adjacent normal tissue. The horizontal lines represent circRNAs expression in the adjacent normal tissues and vertical lines represent circRNAs expression in OSCC tissues. The red plots represent significantly upregulated circRNAs in OSCC tissues, and green plots represent significantly downregulated circRNAs in OSCC tissues. NOR: normal tissues; CAN: cancer tissues; OSCC: oral squamous cell carcinoma; circRNA: circular RNA.

A total of 11,942 circRNA targets, including 1921 known circRNAs and 10,021 novel circRNAs, were detected and defined in 8 pairs of OSCC samples and adjacent normal tissues through high-throughput sequencing (Figure 1). A total of 16 significantly different circRNAs were identified in 8 pairs of samples via high-throughput sequencing, and details regarding these circRNAs are presented in Table 1.

### 3.2. Validation of Hsa_circ_0008309 Expression in the OSCC Tissues

**Figure 2:**
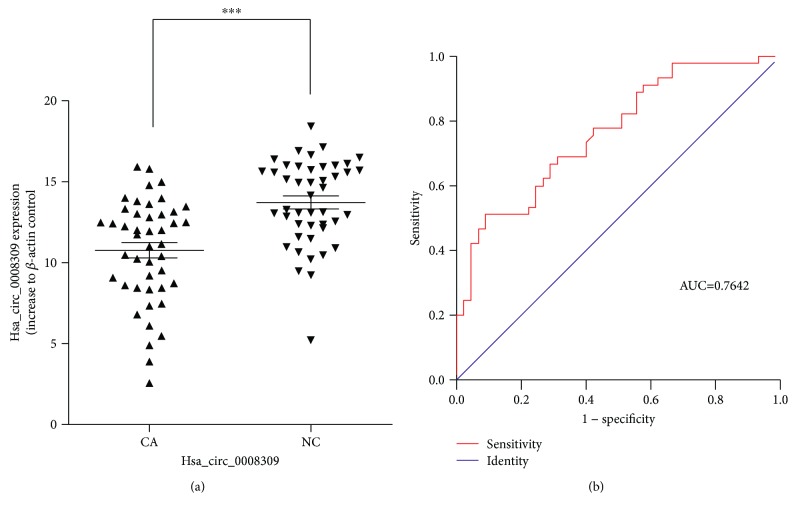
(a) Expression level of hsa_circ_0008309 validated by RT-qPCR in OSCC tissues (*n* = 45) with adjacent normal tissue (*n* = 45). Student's *t*-test (two-tailed) was performed. Data are shown as the mean ± standard deviation. (b) ROC analysis of the expression hsa_circ_0008309 in 45 paired OSCC patients. OSCC: oral squamous cell carcinoma; RT-qPCR: reverse transcription-quantitative polymerase chain reaction; NC: normal tissues; CA: cancer tissues; ROC: receiver-operating characteristic; AUC: area under curve.

The 16 significantly differentially expressed circRNAs in 45 pairs of OSCC samples were analyzed by RT-qPCR. The results demonstrated that hsa_circ_0008309 was significantly downregulated in the carcinoma tissues (Figure 2(a)). According to the human reference genome (GRCh37/hg19) from the Ensembl genome database (http://www.ensembl.org), hsa_circ_0008309 is located at chr2: 225400244-225422573 and the parental gene is Cullin 3 (CUL3). The whole length of the CUL3 gene is 22,329 bp, while the mature transcript of the hsa_circ_0008309 is 312 bp.

### 3.3. The Relationship between Hsa_circ_0008309 and Clinicopathological Characteristics

To confirm the potential diagnostic value of hsa_circ_0008309, the clinicopathological characteristics of the OSCC patients were analyzed with respect to the hsa_circ_0008309 expression level in Table 2. The results found that hsa_circ_0008309 expression was significantly associated with pathological differentiation of OSCC patients. In addition, the diagnostic effect of hsa_circ_0008309 was analyzed via the ROC curve. The area under the ROC curve was 0.7642, indicating the hsa_circ_0008309 is relatively closely associated with OSCC (Figure 2(b)). Taken together, these data indicate that hsa_circ_0008309 may serve as a potential biomarker for the diagnosis of OSCC.

### 3.4. CircRNA-miR-mRNA Network Construction

**Figure 3:**
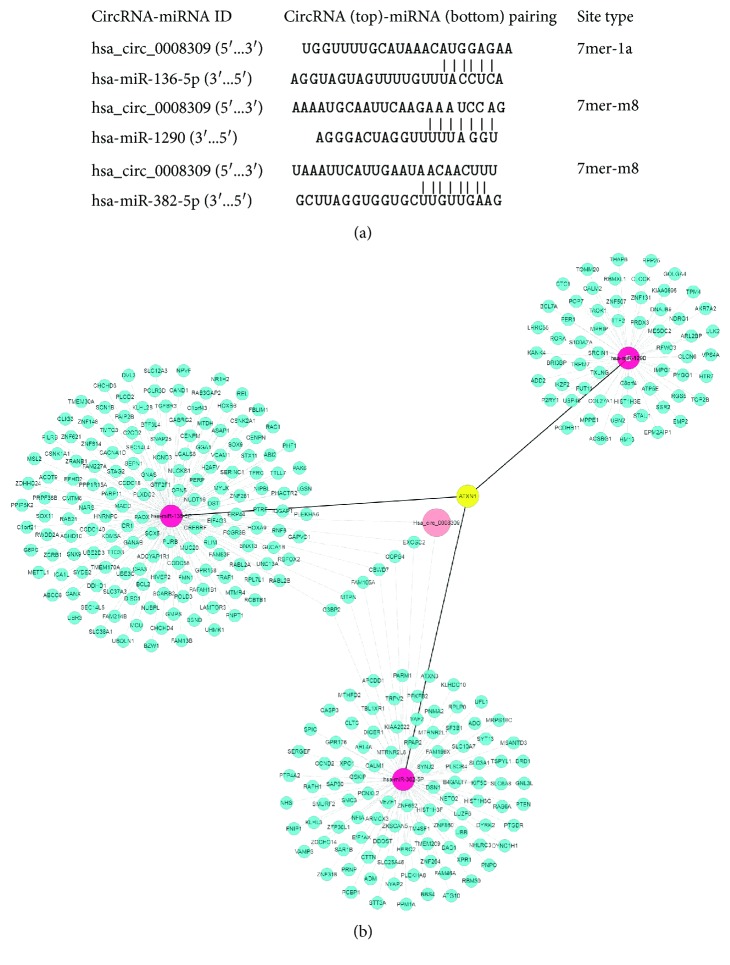
(a) Prediction of hsa_circ_0008309 and miRNAs interactions. (b) CircRNA-miRNA-mRNA network of hsa_circ_0008309. Circ: circular; mi: micro.

Results from the bioinformatics analysis found that hsa_circ_0008309 could, respectively, combine with miR-1290, miR-136-5P, and miR-382-5P (Figure 3(a)). A large number of target genes were identified and some were closely associated with more than one network, as indicated by the bioinformatics analysis (Figure 3(b)). The results demonstrated that ATXN1 was the only gene to be associated with all networks. Therefore, we hypothesize that ATXN1 may be regulated by hsa_circ_0008309 in OSCC.

### 3.5. Validation of Hsa_circ_0008309-miR-136-5P/miR-382-5P-ATXN1 Pathway

**Figure 4:**
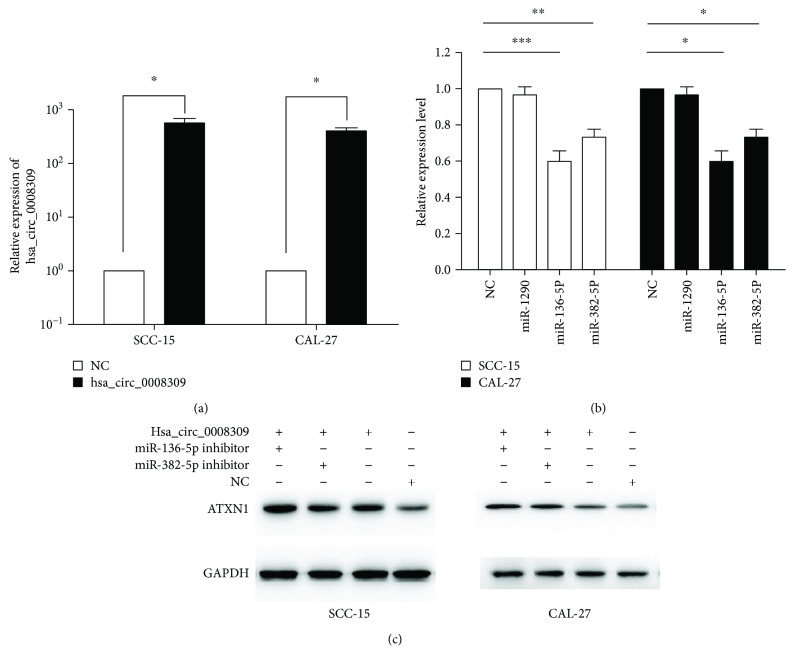
(a) RT-qPCR analysis illustrating the hsa_circ_0008309 expression levels following transfection in SCC15 and CAL27 cell lines. Hsa_circ_0008309 was significantly overexpressed compared with the negative control. A Student's *t*-test (two-tailed) was performed. Data are shown as the mean ± standard deviation, ^∗^*P* < 0.05. (b) Overexpression of hsa_circ_0008309 significantly downregulates the expression of miR-136-5P and miR-382-5P compared with control in SCC15 and CAL27 cell lines by RT-qPCR. One-way ANOVA with Tukey's post-hoc test was performed. Data are shown as the mean ± standard deviation, ^∗^*P* < 0.05, ^∗∗^*P* < 0.01. (c) Western blot analysis shows the ATXN1 protein level in SCC15 and CAL27 cell lines following transfection with hsa_circ_0008309 expression plasmid, miR-136-5P inhibitor plus hsa_circ_0008309 expression plasmid, miR-382-5P inhibitor plus hsa_circ_0008309 expression plasmid and a mock vector. GAPDH was used as an internal control. RT-qPCR: reverse transcription-quantitative polymerase chain reaction; ATXN1: Ataxin 1; miR: microRNA.

After transfection, hsa_circ_0008309 was identified to be overexpressed in the SCC15 and CAL27 cell lines (Figure 4(a)). Compared with the negative control, miR-136-5P and miR-382-5P expression was downregulated in the hsa_circ_0008309 overexpression groups in the SCC15 and CAL27 cell lines (Figure 4(b)). Western blot analysis demonstrated that the ATXN1 protein level was increased when hsa_circ_0008309 expression was upregulated in SCC15 and CAL27 cell lines (Figure 4(c)). These results suggest that hsa_circ_0008309 may regulate the hsa_circ_0008309-miR-136-5P/miR-382-5P-ATXN1 pathway in OSCC cell lines.

## 4. Discussion

CircRNA was previously considered to be a noise from aberrant RNA splicing [16–18]. However, it was recently identified to have the potential as miR sponges and ideal biomarkers in various diseases [19]. In addition, a large number of studies have indicated that various circRNAs were associated with the initiation and progression of cancers [20]; however, there is little evidence regarding the role of circRNA in OSCC.

In the present study, a total of 11,942 circRNAs were identified, demonstrating that circRNAs were in abundant existence in the OSCC samples (*n* = 8). Furthermore, 16 circRNAs were identified to be significantly differentially expressed in the OSCC samples through bioinformatics analysis. The results suggest that circRNA may serve an important role in OSCC, and these significantly differentially expressed circRNAs were subsequently validated in the 45 pairs of OSCC samples. As a result, hsa_circ_0008309 was demonstrated to be significantly downregulated in OSCC tissues (*P* < 0.001). Notably, compared with normal tissues, the hsa_circ_0008309 expression level was revealed to be 2.010811 times in OSCC tissues by high-throughput sequencing. The levels of hsa_circ_0008309 in certain samples were upregulated in the 45 pairs of OSCC tissues; however, the levels of hsa_circ_0008309 in the majority of samples were downregulated. These results indicated that hsa_circ_0008309 expression level of each patient was not consistent and varied between individuals. The expression of hsa_circ_0008309 was downregulated in tumor tissues and was, therefore, more likely to act as a tumor suppressor. The ROC analysis indicated that the hsa_circ_0008309 expression level exhibited a diagnostic role in distinguishing OSCC tissues from adjacent normal tissues. Taken together, we hypothesized that hsa_circ_0008309 has a potential effect on OSCC.

Compared with linear RNA, circRNA has been reported to have an increased number of miR binding sites and may regulate gene expression by acting as miR sponges, thereby regulating linear RNA transcription and protein production [20]. Consequently, the present study examined whether hsa_circ_0008309 had the potential to affect the miRs in OSCC. The bioinformatics analysis illustrated that hsa_circ_0008309 could potentially interact with important miRs including miR-1290, miR-136-5P, and miR-382-5P. In addition, the ATXN1 gene was identified to be strongly associated with these miRs. Therefore, we speculated that hsa_circ_0008309 may regulate the ATXN1 gene by acting as a sponge to these miRs. In order to further explore the role of hsa_circ_0008309, hsa_circ_0008309 was overexpressed in SCC15 and CAL27 cell lines and miR-136-5P and miR-382-5P expression were inhibited, and the ATXN1 protein level was increased when hsa_circ_0008309 was upregulated. ATXN1 was found predominantly in the nuclei of neurons and may function as a transcriptional regulator [21]. Moreover, ATXN1 is a component of the Notch signaling pathway [22], and the Notch signaling reportedly mediates tumor cell migration and invasion induced by low oxygen supply (hypoxia), which is a critical characteristic of solid tumors [23]. Previous studies have demonstrated that a potential link between ATXN1 expression (both upregulation and downregulation) and cancer development in humans could regulate cell proliferation and the epithelial-mesenchymal transition of cells in various cancers [24, 25], but it remains unknown whether ATXN1 acts as a tumor suppressor or an oncogene. Therefore, the ATXN1 might play an important role in the OSCC. According to bioinformatics analysis, the experimental data obtained by the present study suggest that hsa_circ_0008309, a novel emerging circRNA, may regulate ATXN1 by miR-136-5P and miR-382-5P in OSCC cell lines.

In summary, the present study revealed that the OSCC tissues have abundant circRNAs and identified that hsa_circ_0008309 was significantly downregulated in OSCC tissues. Herein is an offered novel approach to explore the role of circRNA by various bioinformatics analyses. Furthermore, the regulatory role of hsa_circ_0008309-miR-136-5P/hsa-miR-382-5P-ATXN1 network was identified in OSCC. These results highlighted the possibility that hsa_circ_0008309 could serve as a potential target for OSCC. The functions and mechanisms of hsa_circ_0008309 in OSCC should continue to be extensively investigated.

## Figures and Tables

**Table 1:** 16 differently expressed circRNAs in the oral squamous cell carcinoma.

CircRNA	Fold change	*P* value	Type	chr	Gene Symbol
Hsa_circ_0008202	−8.00076	0.042153	Exons	1	SPATA6
Hsa_circ_0004491	−3.73247	0.022494	Exons	2	ORC4
Hsa_circ_0008309	2.010811	0.023438	Exons	2	CUL3
Novel_circ_006041	−11.0136	0.035522	Exons	2	FANCL
Novel_circ_007300	−7.53215	0.042153	One_exon	22	MB
Novel_circ_007366	−6.97964	0.036032	Exons	22	FBLN1
Novel_circ_009346	−6.46152	0.042153	Exons	5	EBF1
Novel_circ_006206	−5.84394	0.042153	Exons	2	RMND5A
Novel_circ_008049	−3.74847	0.034611	Exons	3	PHC3
Novel_circ_002578	1.872629	0.030545	Antisense	12	KRT6C
Novel_circ_005748	1.977281	0.035522	Exons	2	MBOAT2
Novel_circ_009749	2.620575	0.036032	Exons	6	PHIP
Novel_circ_003644	2.918808	0.049827	Intergenic	14	NA
Novel_circ_007992	3.151974	0.036032	Exons	3	RNF13
Novel_circ_010219	3.832216	0.035522	Exons	7	FAM126A
Novel_circ_003819	6.455806	0.036032	Exons	15	SPPL2A

**Table 2:** Correlation between hsa_circ_0008309 expression and clinicopathological characteristics in 45 oral squamous cell carcinoma patients.

Characteristics	Total cases (*n* = 45)	*P* value
Sex		0.9828
Male	34	
Female	11	
Age (years)		0.8206
Range (median)	29–78 (54)	
<60	29 (46.655)	
≥60	16 (66.125)	
Site		0.2198
Tongue	25	
Buccal	8	
Gingiva	5	
Floor of the mouth	7	
Clinical stage		0.2682
0&I + II	19	
III + IV	26	
Pathological differentiation		0.0386^∗^
Well	19	
Moderately	20	
Poorly	6	
LNM		0.3176
Yes	17	
No	28	

^∗^ *P* < 0.05. LNM: lymph node metastasis.

## Data Availability

The data used to support the findings of this study are available from the corresponding author upon request.
